# Error in Captions of Figures 1 and 2

**DOI:** 10.1001/jamanetworkopen.2024.19798

**Published:** 2024-05-31

In the Original Investigation titled “Comparative Effectiveness of Different Treatment Pathways for Opioid Use Disorder,”^1^ published February 5, 2020, the expansion of “CE” in the captions of Figures 1 and 2 was changed from “continuing education” to “continuous enrollment.” This article has been corrected.^1^

## References

[1] Wakeman SE, Larochelle MR, Ameli O, . Comparative effectiveness of different treatment pathways for opioid use disorder. JAMA Netw Open. 2020;3(2):e1920622. doi:10.1001/jamanetworkopen.2019.20622 32022884 PMC11143463

